# Role of Progesterone Receptor Polymorphisms in the Recurrent Spontaneous Abortions: Indian Case

**DOI:** 10.1371/journal.pone.0008712

**Published:** 2010-01-14

**Authors:** Meka Aruna, Theeya Nagaraja, Sadaranga Andal, Surapaneni Tarakeswari, Pisapati V. S. Sirisha, Alla G. Reddy, Kumarasamy Thangaraj, Lalji Singh, B. Mohan Reddy

**Affiliations:** 1 Molecular Anthropology Group, Biological Anthropology Unit, Indian Statistical Institute, Hyderabad, India; 2 Lakshmi Fertility Clinic and Research Centre, Pogathota, Nellore, Andhra Pradesh, India; 3 Fernandez Hospital, Hyderabad, India; 4 Centre for Cellular and Molecular Biology, Hyderabad, India; Health Canada, Canada

## Abstract

**Background:**

We attempt to ascertain if the 3 linked single nucleotide polymorphisms (SNPs) of the Progesterone Receptor (PR) gene (exon 1: G 1031 C; S344T, exon 4: G 1978 T; L660V and exon 5: C 2310 T; H770H) and the PROGINS insertion in the intron G, between exons 7 and 8, are associated with Recurrent Spontaneous Abortion (RSA) in the Indian population.

**Methodology/Principal Findings:**

A total of 143 women with RSA and 150 controls were sequenced for all the 8 exons looking for the above 3 linked SNPs of the PR gene earlier implicated in the RSA, as well as for any new SNPs that may be possibly found in the Indian population. PROGINS insertion was screened by electrophoresis. We did not find any new mutations, not observed earlier, in our population. Further, we did not find significant role of the *2 allele (representing the mutant allele at the three SNP loci) or the T2 allele (PROGINS insertion) in the manifestation of RSA. We also did not find an LD pattern between each of the 3 SNPs and the PROGINS insertion.

**Conclusions/Significance:**

The results suggest that the PR gene mutations may not play any exclusive role in the manifestation of RSA, and instead, given significantly higher frequency of the *2 allele among the normal women, we surmise if it does not really confer a protective role among the Indian populations, albeit further studies are required in the heterogeneous populations of this region before making any conclusive statement.

## Introduction

Recurrent Spontaneous Abortions (RSA) is defined as repeated occurrence of 3 or more miscarriages before 24^th^ week of gestation [1]. The modern definition, however, is the spontaneous loss of 2 or more consecutive pregnancies before 20 weeks of gestation [2], [3]. Implantation of the embryo is a critical event in pregnancy. In humans, peri-implantation pregnancy loss contributes to more than 20% of unexplained infertility. Deficient hormone levels result in aberrant growth and support of the uterine lining making it un-ideal for implantation.

Progesterone, a 21 carbon steroid hormone, mainly produced in the ovaries, placenta, brain and the adrenal glands, is required for the maintenance of pregnancy and treatment with progesterone supplementation was observed to prevent abortions. It is mainly involved in the female menstrual cycle, pregnancy and embryogenesis in most mammalian species. It stimulates and regulates various functions - i) helps in preparing the body for conception and pregnancy (implantation of the embryo, promoting uterine growth and suppressing myometrical contractility) [4]–[6] ii) acts as an anti-inflammatory agent and regulates the immune response [7] and iii) regulates estrogen levels and thus prevents endometrial cancer.

Progesterone receptor (PR) mediates the physiologic effects of progesterone. The PR gene uses separate promoters and translational start sites to produce 2 isoforms, PRA and PRB, the only difference between the two being an additional 165 amino acids present in the N terminus of PRB. The human progesterone receptor (hPR) gene was localized to 11q22–q23 [8] and spans over 90 kb containing eight exons [9]. The open reading frame corresponds to a protein of 933 amino acids with a molecular weight of 98,868 Da [10].

Three linked single nucleotide polymorphisms (SNPs) (exon 1: G 1031 C; S344T, exon 4: G 1978 T; L660V and exon 5: C 2310 T; H770H) in the PR gene were found to be associated with RSA [11]. The SNP in the exon 1 is reported to be apparently linked to the SNPs in exons 4 and 5 [11], which are in turn in linkage disequilibrium (LD) with PROGINS, a 306 base pairs (bp) insertion of PV/HS-1 *Alu* subfamily in intron G, between exons 7 and 8 in the codifying region of the hormone binding domain [11], [12], [13]. However, contrary to the expectation, Kurz et al. [14] suggest that PROGINS is not associated with idiopathic RSA. Thus, only two studies dealt directly with the association of PR mutations with RSA, one dealing with PROGINS insertion and other with the 3 SNPs. Polymorphic variants of hPR gene have been implicated in implantation failure [15], [16]. There were also studies which did not show association of PR mutations in implantation failure [17] and preterm birth [18]. To the best of our knowledge, most of the earlier studies on the role of the PR mutations in adverse reproductive outcomes were in isolation not in conjunction with the PROGINS insertion. Further, LD between the PROGINS and the SNPs has been assumed rather than empirically tested. Thus, the earlier studies concerning the role of hPR gene in RSA have been largely inconclusive.

Given that most studies hitherto undertaken were on the Caucasian populations, it is not known if ethnicity has any role in the observed pattern of association of PR polymorphisms with adverse pregnancy outcomes. There were no studies on the role of these polymorphisms vis-à-vis RSA in the Asian populations, especially from India, which is known for its unique population structure characterized by strictly endogamous castes and tribes. In view of this, we attempt to ascertain if the 3 linked SNPs of the hPR gene and the PROGINS insertion are associated with RSA in the relatively homogenous Indian sample. We also attempt to explore if we can find any novel SNPs in hPR gene that can be implicated in RSA.

## Results

**Figure 1:**
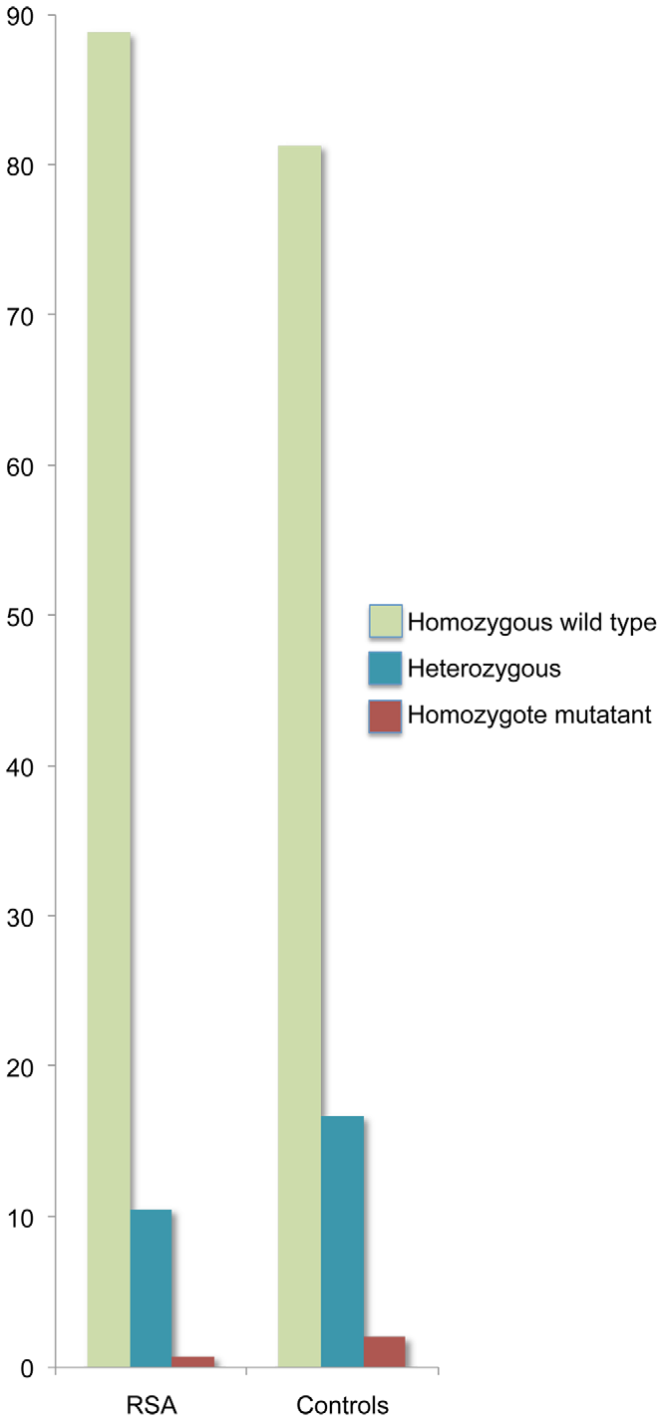
Genotype distribution of the PR mutations; (*1) wild type (G1031, G1978, and C2310) and (*2) mutant allele.

We did not find any new SNPs in hPR gene, other than the 3 found earlier. The three SNPs found in the PR gene – exon 1: G 1031 C, S344T; exon 4: G 1978 T, L660V; exon 5: C 2310 T, H770H – co-inherit. The genotype frequencies of the homozygotes for wild type allele (*1), heterozygotes (*1/*2) and homozygotes for the rarer mutant allele (*2) in the RSA women were observed to be 127 (88.8%), 15 (10.5%) and 1(0.7%), respectively, as compared to 122 (81.3%), 25 (16.7%), and 3(2%) in controls (Figure 1). The genotype frequencies were not significantly different between RSA and control women (χ^2^ = 3.44, df = 2, p = 0.18).

The allele frequencies (Table 1) were significantly different between the cases and the controls in the pooled samples (χ^2^ = 3.75, df = 1, p = 0.05) as well as between the RSA women with ≤3 abortions and controls (χ^2^ = 4.88, df = 1, p = 0.03). However, this significance is due to an increased frequency of the *2 allele among the controls, not among the RSA women. Similar trend was seen when allele frequencies were calculated for the patients from LFC and FMH and for primary and secondary aborters separately. Although significant χ^2^ values were observed to suggest association in the LFC data (χ^2^ = 4.24, df = 1, p = 0.04), as well as for the primary aborters (χ^2^ = 3.85, df = 1, p = 0.05) the perceived risk allele (*2), contrary to the expectation, is observed in higher frequency among the controls, suggesting probably a protective role of this allele. Further analysis was carried out to check, if this trend can be statistically validated. Logistic regression was performed considering a possible protective role for the *2 allele. Although the odds ratio (OR) for the pooled RSA sample and the controls was only marginally significant (p = 0.056), OR for the RSA women with 2–3 abortions and the controls was significant (p = 0.03), suggesting a protective role for the allele (Table 2).

**Table 1:** Allele frequencies of the *1 and the *2 alleles.

	Allele Freq							
PR mutation	RSA pooled (n = 286)	RSA (n = 250) (2–3 abortions)	RSA (n = 36) (≥4 abortions)	RSA (n = 166) (LFC)	RSA (n = 120) (FMH)	RSA (n = 260) (1° aborters)	RSA (n = 26) (2° aborters)	Controls (300)
*1	0.941	0.948	0.889	0.952	0.925	0.942	0.923	0.897
*2	0.059	0.052	0.111	0.048	0.075	0.058	0.077	0.103

Allele Frequencies of the *1 and the *2 alleles in the RSA women (pooled, 2–3 abortions and 4 or more abortions) and the controls, RSA women from LFC and FMH and in the primary (1°) and secondary (2°) aborters.

RSA women and controls (**χ^2^** = 3.75, DF = 1, p = 0.05). RSA women (2–3 abortions) and controls (**χ^2^** = 4.88, DF = 1, p = 0.03). RSA women (≥4 abortions) and controls (**χ^2^** = 0.02, DF = 1, p = 0.88).

RSA women (LFC) and controls (**χ^2^** = 4.24, DF = 1, p = 0.04). RSA women (FMH) and controls (**χ^2^** = 0.80, DF = 1, p = 0.37). RSA women (LFC) and (FMH) (**χ^2^** = 0.90, DF = 1, p = 0.34). RSA women (1°) and controls (**χ^2^** = 3.85, DF = 1, p = 0.05). RSA women (2°) and controls (**χ^2^** = 0.18, DF = 1, p = 0.67). RSA women (1°) and (2°) (**χ^2^** = 0.16, DF = 1, p = 0.69).

**Table 2:** Odds ratio for the *2 allele in RSA pooled samples and in the 2–3 abortions.

	B±S.E.	p-value	OR	95.0% C.I. for OR	
				Lower	Upper
Pooled RSA cases Vs controls	0.601±0.314	0.056	1.824	0.986	3.374
RSA (2–3 abortions) Vs controls	0.742±0.342	0.030	2.101	1.074	4.109

Statistical power (1-β) of these results was computed using G*Power 3.1. Given the large sample (2N), the power obtained was significant at 80% for pooled RSA women (286) and controls (300) and 87% for RSA women with 2–3 abortions (250) and controls (300), conferring fair degree of reliability to the findings of this study.

**Figure 2:**
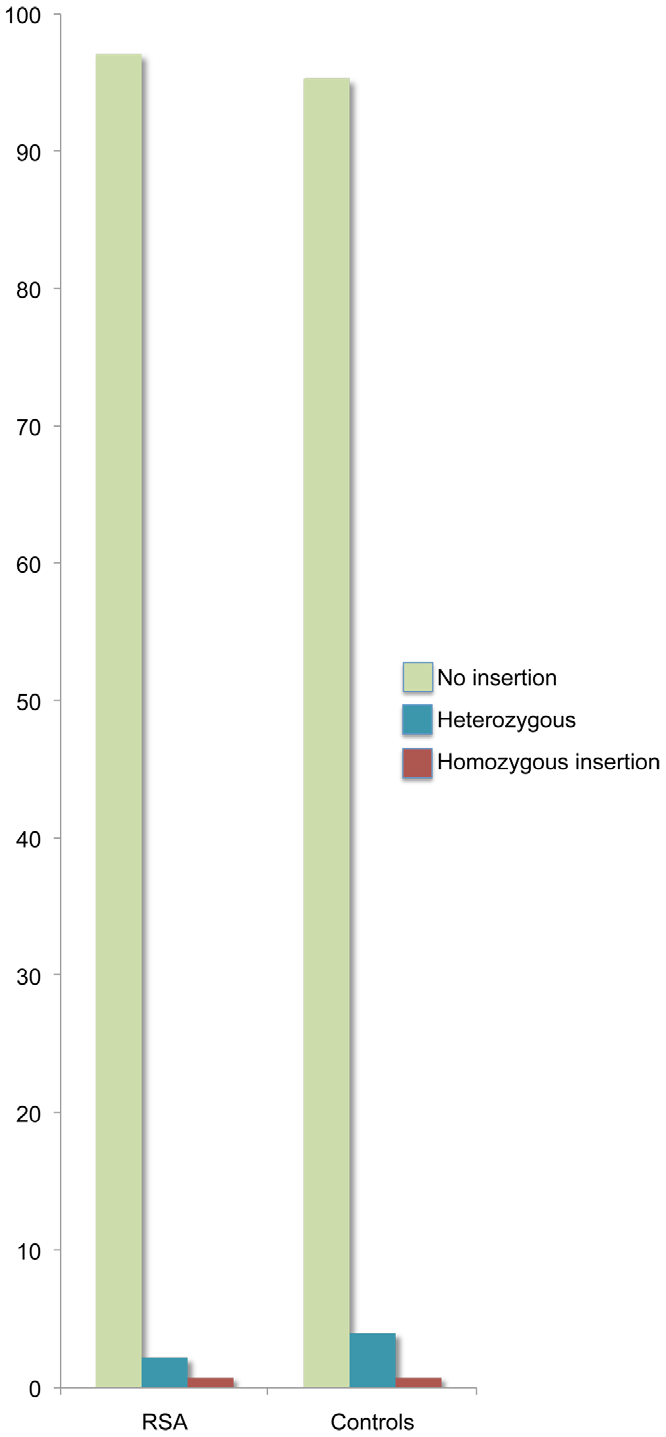
Genotype distribution of PROGINS insertion; (T1) wild type (no insertion) and (T2) insertion allele.

The analyses of PROGINS *Alu* insertion revealed genotypic frequencies of 133 (97.1%) homozygous wild type (T1/T1), 3 (2.2%) heterozygous (T1/T2) and 1 (0.7%) homozygous PROGINS insertion (T2/T2) among the RSA women as compared to the frequencies of 143 (95.3%), 6 (4%) and 1 (0.7%) among the controls (Figure 2). The genotype frequencies were not found to be significantly different between RSA and control women (χ^2^ = 1.05, df = 2, p = 0.59). Allele frequencies for the T1 and T2 alleles were observed to be 0.982 and 0.018 and 0.973 and 0.027, respectively for the RSA and control women. The difference in the allele frequencies between RSA and control women was not significant in either the pooled sample or when RSA women with different number of abortions are separately considered.

**Figure 3:**
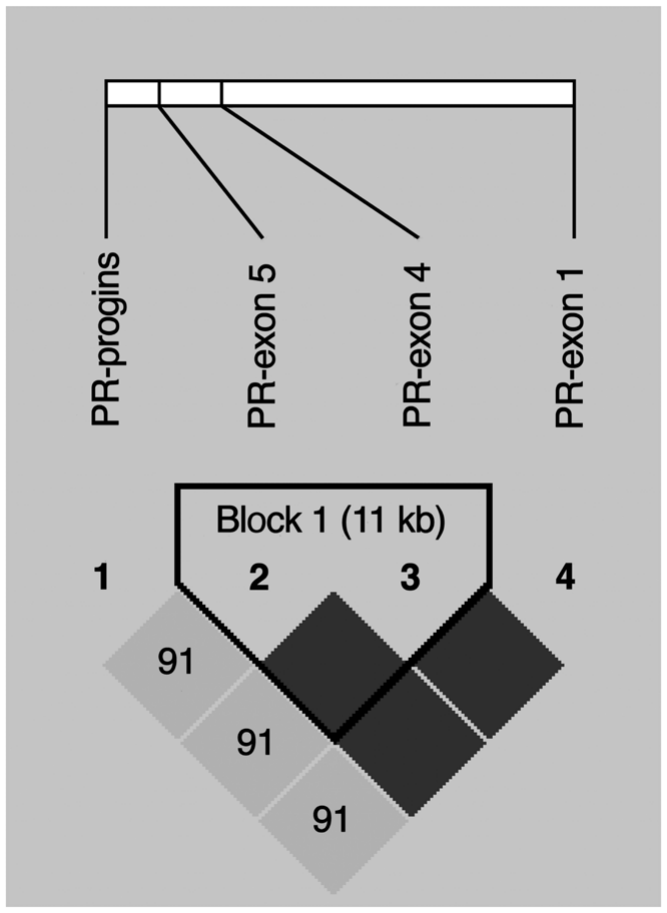
LD plot showing the confidence bounds color scheme where dark grey suggests strong evidence of LD and light grey suggests an uninformative status of LD. The analysis reveals that exons 4 and 5 inherit as an LD Block.

The mutant alleles considered together at the three SNP sites (*2 allele) and the PROGINS insertion (T2 allele) are reported to be in LD [11], [12], [13]. LD analysis was carried out using Haploview version 4.1 [19] to check if the SNPs in the coding region and the insertion polymorphism in the intron G were in LD in our sample. Given the distance between the SNPs in exons 5 and 1 (76kb), our haploview analysis revealed an LD block (Figure 3) consisting of only the two SNPs in exons 4 and 5 (separated by 11 kb), even though the D′ value and the correlation coefficient for all pairs of comparisons between the three SNPs (i.e., between exons 5 and 4, exons 5 and 1, exons 4 and 1) is 1. The LD analysis suggests that the exons 1, 4 and 5 do not show complete LD with the PROGINS.

## Discussion

Progesterone receptor mediated effects play a critical role in female reproduction [5]–[7], [20]–[22]. We investigated mutations of the progesterone receptor gene and observed an increased frequency of the *2 allele in the controls (10.3%) when compared to the RSA women (5.9%). The frequency of 2.7% of the T2 allele in the controls in our study is relatively close to the frequency of 5.5% among the normal women from Hyderabad [23]. In our study, 3 women in the control group were homozygous for the *2 allele, yet they had no abortions suggesting that even the homozygous mutations are not sufficient to cause RSA. Haploview analysis suggests that even though the three SNPs show a D′ of 1, the LD block consists of the SNPs in the exons 4 and 5 only. Based on our genotyping results, we find that the insertion (T2 allele) is seen only among the individuals with at least one copy of the *2 allele suggesting a possible association between the *2 allele and the T2 allele, which is in concordance with the results of Haploview analysis revealing a partial LD.

We did not find any significant increase in the frequency of either the *2 allele or the T2 allele among RSA women in our study of the Indian population and contrary to the expectations, we find a higher frequency of the *2 allele in the controls. Therefore, our results based on a relatively larger and more homogeneous Indian sample suggest that PR gene mutations may not play a significant role in the manifestation of RSA and, instead, prompt one to surmise if the *2 allele does not really confer a protective role among the Indian populations. Our results are not in agreement with previous reports of associations between progesterone receptor mutations and adverse reproductive outcomes including unexplained infertility [15], implantation failure after IVF/ET [16] and unexplained RSA [11]. Association between PR polymorphisms and adverse pregnancy outcomes was both reported [11], [15], [16] and refuted [14], [17], [18]. The discrepancy in the results could be due to multifactorial etiology as there may be other genes acting in conjunction with the PR, each with relatively small effect. To be able to detect the relatively small contribution of PR polymorphisms one would require very large sample size which has not been the case in most of the studies. Further, the effect of progesterone receptor is minimal after 6 weeks of gestation when compared with during the process of implantation [24]. Most RSA cases that one enrolls are usually much later than 6 weeks of gestation and therefore, PR mutations may not be the right candidates. Heterogeneity in study approaches may also contribute to this inconsistency.

To the best of our knowledge, ours is by far the largest sample as far as the studies of PR mutations in RSA are concerned. For example, the studies concerning unexplained infertility [15] and RSA [11] that yielded positive association with PR mutations were based on only 26 and 42 cases. Even the studies that yielded negative results with reference to RSA [14], recurrent implantation failure [17] and preterm birth [18] were with 125, 66 and 78 cases, respectively, whereas we used 143 cases and 150 controls. Given the large sample size, the statistical power of our study should be relatively higher. The 87% of power obtained with reference to odds ratio probably bears testimony to this, conferring fair degree of reliability to the findings of our study. We strongly believe that the ethnic differences in the nature of genetic predisposition to different complex genetic disorders could also lead to inconsistency in the pattern of association in our population, as has also been reported earlier with reference to other complex disorders in the Indian populations [25]. Thus the incongruent nature of our findings in the Indian population concerning the role of PR gene mutations in RSA Vis-a-Vis the non-Indian populations adds to the ever increasing body of evidence on the novel patterns of genetic predisposition of Indian populations to different complex diseases, underlining the importance of unique Indian population genetic structure.

Despite the crucial role that progesterone plays in the maintenance of the pregnancy the genetic analysis of PR gene did not provide formidable evidence towards its association with RSA, either in the western or Indian populations so as to attach any prognostic value for RSA. However, since progesterone is essential for the development of a receptive endometrium, it is necessary to also consider: progesterone levels (as treatment with Dydrogesterone or Progesterone helps in prevention of RSA [26]–[30]), PR expression levels (reduced levels have been reported in RSA women [31]–[33]), its transcription, its relation with estrogen receptor (ER) and its role in immune modulation along with the gene mutations in populations of diverse ethnic and geographic backgrounds before completely ruling out the role of PR in the manifestation of RSA. On the other hand, our results also suggests a probable protective role of the *2 allele. Since this study is the first of its kind in the populations of this region, one should await results from more populations of diverse ethnic and geographic backgrounds before making any conclusive statement on the role of PR in RSA, protective or otherwise.

## Materials and Methods

### Samples

For the present study, samples were collected from a relatively homogenous Telugu population from Andhra Pradesh, India. Women with RSA were recruited from two different hospitals - Lakshmi Fertility Clinic (LFC) in the suburban Nellore town and Fernandez Maternity Hospital (FMH) in metropolitan Hyderabad. These two hospitals not only represent two different socio economic strata of the patients, but are also separated geographically by about 500kms. Control samples were collected from Hyderabad, Nellore and nearby villages so that they broadly represent matched ethnic and socio-economic backgrounds with that of the cases. This framework facilitates to test if the results are consistent between the socioeconomically contrasting but genetically somewhat homogenous samples; screening a number of autosomal STR markers from 27 Telugu populations (castes and tribes) from different socioeconomic strata, Reddy et al [34] observed fair degree of homogeneity across the socioeconomic strata of the populations which form a compact cluster when compared to the populations of the other regions and linguistic groups.

Peripheral blood samples (3–5 ml) were collected in EDTA coated vacutainers from 143 women with RSA of unknown etiology. Of these 83 were from LFC while remaining 60 were from FMH. All RSA women, with the mean age of 26 yrs (range 18–37 years of age) and with number of miscarriages ranging from 2 to 9, had regular menstrual cycles and were healthy. Karyotypes were normal. The women underwent careful clinical examinations, as well as analysis of tissue antibodies and auto-antibodies as prescribed by their doctors. Women normal for the above tests as well as for the blood glucose and thyroid stimulating hormone concentrations were enrolled for the study. A possible infectious etiology was also ruled out by assessing the reports of the microbiological cultures of the samples obtained from the cervix and uterine cavity. A total of 130 women had no previous children (primary aborters) while 13 had one or two children before the consecutive miscarriages (secondary aborters). Our control group consisted of 150 healthy women with no history of abortions and at least one live born child. Women in the control group were aged between 18–45 years. Blood samples from the cases as well as the controls were collected with written informed consent and we had obtained approval for this study from the Indian Statistical Institute Review Committee for Protection of Research Risks to Humans.

### Polymerase Chain Reaction and DNA sequencing

**Figure 4:**
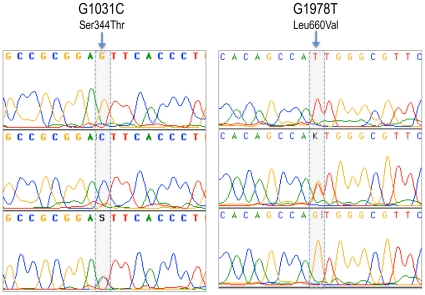
Electropherograms of the two SNPs of hPR gene in exon 1 (G1031C) and exon 4 (G1978T).

**Figure 5:**
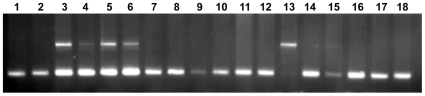
Gel picture showing bands for homozygotes (T1) allele, heterozygotes (T1/T2), and homozygote (T2) allele run on 2% agarose. Lane 3, 4, 5, and 6 are heterozygotes, and lane 13 is homozygous for the T2 allele and the remaining are homozygous for the T1 allele.

DNA was isolated from the above samples following Sambrook et al., [35] protocol. PCR amplification of all the 8 exons of PR gene was carried out as per Schweikert et al. [11] and the PROGINS *Alu* insertion analysis was carried as per Gomes et al., [36]. Reactions were carried out in an ABI Gene Amp PCR system 9700. Cycle Sequencing of PCR products of the 8 exons was carried out with either the forward or the reverse primers using the Big-Dye Terminator ready reaction kit (Perkin Elmer) depending on the position of the mutation and analyzed on an ABI 3730 automated DNA Analyzer (Applied Biosystems) (Figure 4). The PROGINS amplification products were loaded in a 2% agarose gel, stained with ethidium bromide (1µg/ml), and electrophoresis was carried out at 100 volts for 30 minutes, in 0.5× TAE buffer (Figure 5).

### Statistical analysis

Allele frequencies were calculated by gene counting method for the case and control samples, women with ≤3 and ≥4 abortions, RSA women from LFC and FMH and primary and secondary aborters separately. χ^2^ analysis and logistic regression were carried out using SPSS. G*Power was employed to detect the power of the study. Linkage disequilibrium estimates, for the three SNP loci and the PROGINS insertion, were calculated using Haploview 4.1 software.
